# Effects of Isolated Soy Protein Supplementation Combined with Aerobic Exercise Training on Improving Body Composition, Anthropometric Characteristics and Cardiopulmonary Endurance in Women: A Pilot Study

**DOI:** 10.3390/ijerph182211798

**Published:** 2021-11-10

**Authors:** Fang Li, Ying-Ting Hsueh, Yi-Ju Hsu, Mon-Chien Lee, Chun-Hao Chang, Chin-Shan Ho, Chi-Chang Huang

**Affiliations:** Graduate Institute of Sports Science, National Taiwan Sport University, Taoyuan City 333325, Taiwan; lif848332@gmail.com (F.L.); 1070201@ntsu.edu.tw (Y.-T.H.); ruby780202@ntsu.edu.tw (Y.-J.H.); 1061304@ntsu.edu.tw (M.-C.L.); 1031301@ntsu.edu.tw (C.-H.C.)

**Keywords:** isolated soy protein, aerobic exercise training, body composition, anthropometric characteristics, cardiopulmonary endurance

## Abstract

Today, women are concerned with health promotion but also with improvements in body weight and shape. The purpose of this study was to investigate the effects of aerobic exercise training (AET) combined with isolated soy protein (ISP) supplementation on the body composition, anthropometric characteristics, and cardiopulmonary endurance of women. The qualified subjects were randomly assigned to AET or AET + ISP groups. Women in the AET + ISP group were given an ISP-rich supplement (40 g/day) 5 days a week for 8 weeks; those in the AET group were given the same amount of water in an identical manner. All women received 60 min of AET twice a week for 8 weeks at an intensity of 40–65% heart rate reserve (HRR) and their body composition, anthropometric characteristics, and physical fitness were measured one week before and after the 8-week AET class. A total of 16 subjects (age: 36.13 ± 5.76 years) completed the study and were included in the dataset. The results of this study show that the AET + ISP group obtained greater reductions in body weight (effect size = 0.99), body mass index (BMI, effect size = 1.04), percentage body fat (PBF, effect size = 1.18), circumferences (waist and hip, all effect sizes > 0.8), and greater gains in the percentage lean body mass (PLBM, effect size = 0.89), compared with the AET group, without significant differences in 20 m multi-stage shuttle run test (20 m MST). We conclude that there is a trend for the consumption of ISP following AET to improve the body composition and anthropometric characteristics in women, compared with those who received the same AET without ISP supplementation.

## 1. Introduction

The effects of consuming plant-based isolated soy protein (ISP) are related to health and physical function; it provides most of the essential amino acids for protein synthesis in the body [1,2]. ISP exerts antioxidant effects in humans, which are associated with a reduction in the incidence of cancer, type 2 diabetes, and cardiovascular disease [3,4,5,6]. ISP intake can reduce the levels of low-density lipoprotein cholesterol, total cholesterol, triglycerides, and breast cancer mortality and improve vascular elasticity and glycemic control [3,7,8,9,10,11]. Additionally, small amounts of dietary protein reduce the breakdown of muscle protein, whereas large amounts of protein supplementation stimulate the synthesis of muscle protein [12]. Protein supplementations allow for higher protein intake, and consuming adequate ISP can result in decreased body fat and weight; thus, they can be used by exercising individuals who require more protein to maintain lean body mass and accelerate fat loss [4,12,13,14,15].

Aerobic exercise is beneficial for improving basal myofibrillar protein synthesis, capillary density, and muscle quality and preventing cardiovascular disease, and decreasing the levels of visceral fat, low-density lipoprotein cholesterol, and glycemia [16,17,18,19]. For women, aerobic exercise can also reduce the risk of developing breast cancer, increase hemoglobin, hematocrit, red cell, and platelet counts, and decrease prolactin, estradiol, and progesterone levels, thereby contributing to relieving fatigue, confusion, and most premenstrual symptoms [20,21,22,23,24]. Aerobic exercise for a prolonged period (>6 weeks) has been observed to reduce body weight, fat mass, waist and hip circumference, and result in gains in aerobic power [25,26,27]. Furthermore, previous studies have demonstrated that combining aerobic and strength training was more effective in improving body composition, strength, and cardiovascular fitness than aerobic training alone [28,29], but no differences were found in metabolic syndrome improvement [30,31]. For most adults, a combination of moderate (e.g., 40–59% HRR) and vigorous (e.g., 60–89% HRR) intensity aerobic exercise for ≥3–5 days/week, 20–60 min per day is recommended by the American College of Sports Medicine (ACSM) [32].

Shortly after exercise, consuming 20–40 g of protein is sufficient to maximally stimulate muscle and albumin protein synthesis [33,34]. Since exercise commonly induces muscle protein breakdown, enough protein post exercise can provide the body with the amino acids needed to repair and rebuild muscle, attenuate exercise-induced muscle damage, and enhance muscle recovery, thus promoting exercise performance and physical health [2,35]. The positive effects of ISP intake following resistance exercise training on muscle protein synthesis, fatigue reduction, and muscle mass maintenance and gains are well documented [2,36,37], but there are few studies concerning the effects of ISP supplementation following AET on health promotion. Although our previous study confirmed that protein supplementation after AET can improve the exercise performance and body composition of mice [38], their body composition is not similar enough to be able to draw this conclusion for humans. Therefore, it is hard to know if protein supplementation post AET could promote human health, especially for women.

Today, women are preoccupied with health promotion but also improvements in body weight and shape and thus represent a large proportion of consumers in the nutritional supplementation market [39,40]. Therefore, the primary aim of this study was to investigate the effects of ISP supplementation combined with AET on improving body composition, anthropometric characteristics, and cardiopulmonary endurance in women. We hypothesized that consumption of ISP in women undergoing an AET program for 8 weeks would induce greater improvements in these physiological characteristics compared with AET only, thus further contributing to promoting physical health.

## 2. Materials and Methods

### 2.1. Study Design

Anthropometric characteristics and body composition measurements were determined for all the qualified subjects, who were also subject to a physical fitness test and were randomly assigned to one of the two intervention groups: AET with water supplement (AET group) or AET with an ISP-rich supplement (AET + ISP group). High-purity and high-quality ISP-rich supplements certified by Societe Generale de Surveillance (SGS Taiwan, New Taipei City, Taiwan) and uncontaminated by heavy metals, herbicides, or insecticides were provided by Bestjet Biotechnology Co., Ltd. (New Taipei City, Taiwan); each 20 g package contained 11.6 g of high-purity protein, the compositions of which are shown in Table 1. All subjects received the same AET regimen. The study complied with the Declaration of Helsinki and was conducted in accordance with the International Conference on Harmonization and Good Clinical Practice (ICH-GCP) guidelines [41]. The study was approved and reviewed by the Landseed Hospital International Institutional Review Board (Taoyuan, Taiwan; LSHIRB No. 18-018-A2). Before starting the experiment, the researchers explained the experimental process in detail, and the experiment began after the subjects signed the consent form.

### 2.2. Subjects

A total of 16 women aged 25–46 years completed this study (AET, N = 8; AET + ISP, N = 8). No baseline differences (*p* > 0.05) in the body composition, anthropometric characteristics, and physical fitness indicators were observed between AET and AET + ISP groups (Table 2). Women in the AET + ISP group were given an ISP-rich supplement (40 g/day) 5 days a week for 8 weeks and drank 40 g of ISP supplement powder dissolved in 400 mL of water within 30 min after each AET class. Subjects in the AET group were given the same amount of water in an identical manner. During the experimental intervention, nutritional supplementations other than ISP were not allowed. The subjects were instructed to maintain their normal lifestyles and eating habits but not to ingest any food or drinks containing caffeine; their diets were recorded and then analyzed by professional dietitians using the Nutritionist Professional software (E Kitchen Business Co., Taipei City, Taiwan). Eligible subjects were recruited using an open, independent, and random method by posting advertisements in public spaces. Inclusion criteria were healthy women with sedentary behavior [42], aged 20–55 years, without any regular exercise habits. Individuals with cardiovascular disease, hypertension, asthma, or a soy allergy, and those who had bone, muscle, or nerve damage were excluded.

### 2.3. Anthropometric Measurements and Body Composition

Measurements of the chest, arm, waist, hip, thigh, and calf circumferences and the tricep, suprailiac, and thigh skinfold thickness were performed by the same professional using a tape measure and skinfold caliper (British Indicators Ltd., St. Albans, Herts, UK) and in accordance with the ACSM’s guidelines for exercise testing and prescription [32]. The body weight, PBF, and muscle mass were measured using the TANITA body fat scale (BC-565, Tanita Co., Tokyo, Japan). BMI was calculated using the formula of body weight (kilograms) divided by height (meters) squared.

### 2.4. Physical Fitness Test

Assessment of physical fitness included tests for muscular endurance (1 min sit-up), flexibility (sit and reach), upper body muscular endurance (1 min push-up), and cardiopulmonary endurance (20 m MST) [32]. Physical examinations were performed one week before and after the 8-week classes. The 1 min sit-up test was performed in pairs of two partners. During the test, the person to be tested lay down flat on a pad with both hands crossed on the chest and both knees bent at a 90° angle. The other partner held the ankles of the test person. When signaled to start by the partner, the test person sat up until their elbows touched their thighs. These steps were repeated for 1 min, and the number of performed sit-ups was recorded. A higher number of sit-ups indicated stronger endurance of the abdominal muscles.

For the sit-and-reach test, a ruler was placed on the ground between the two legs toward the feet, which were placed at the 25 cm mark. The hands were placed on top of each other with the middle fingertips overlapped. The hands then reached slowly forward with the legs straight. At the point of the greatest reach, the distance beyond the 25 cm mark was recorded. The test included a trial and two test runs. The test run with the longest reach distance was recorded.

The 1 min push-up test is administered with women starting in the modified “knee push-up” position [32]. The arms were placed one-shoulder-width apart and extended at a right angle to the body. The body was lowered until the elbow formed a 90° angle and then pushed up until the arms were straight. These steps were repeated, and the number of push-ups performed in 1 min was recorded.

The validity of the 20 m MST for assessing a subjects’ cardiopulmonary endurance has been examined [43]. The 20 m MST consists of continuous running back and forth between two cones placed 20 m apart. The subjects started running at 8.5 km/h and then increased their speed by 0.5 km/h per minute. The subjects continued to run until they were exhausted. The distance covered in the shuttle runs was recorded and represented the test result [44].

### 2.5. Aerobic Exercise Training

According to ACSM guidelines for exercise testing and prescription, moderate exercise intensity corresponds to 40–59% HRR, 64–76% HRmax, and 12–13 rating of perceived exertion (RPE, 6–20 scale), whereas vigorous exercise intensity corresponds to 60–89% HRR, 77–95% HRmax, and 14–17 rating of RPE [32]. In this study, all women received 60 min of AET class twice a week for 8 weeks at the intensity of 40–65% HRR. Each 60 min exercise class included 10 min of warm-up, 5 min of muscle exercise, 5 min of stretching, and 40 min of the main exercise. The main exercise included boxing (jap, cross, uppercut, and hook), kickboxing (front push kick and uppercut squat reach), karate (straight punch and front kick), Thai boxing (ascending elbow and knee), and martial arts (elbow strike and knee strike); these exercises were intended to improve the hitting and kicking power [45]. During the 10 min warm-up period, all muscle motions required for the exercise were performed. The main exercise started with leg endurance training, followed by training to improve the arm strength and movement speed and to enhance cardiorespiratory endurance. The 5 min muscle exercise was intended to strengthen the abdomen, back, and hip core muscles. The 5 min stretching exercise (arm circles, hip flexor stretches, spinal flexion and extensions) was employed for the enhancement of joint and muscle flexibility. The heart rate was monitored in real time during exercise using a sports watch (UTA S2, Changjiang Technology Co., Beijing, China). After each AET class, subjects were asked to assess their own physical condition according to the RPE protocol. All AET classes were led by the same coach.

### 2.6. Sample Size Calculation

G*Power software (version 3.1.9.6, Universität Kiel, Kiel, Germany) was employed to calculate the sample size [46]. According to the analysis of covariance (ANCOVA), in terms of fixed effects, main effects, and interactions, with the effect size of 0.8, alpha = 0.05, power = 0.80, numerator = 1, and two groups with one covariate, the total sample size needed is 15 for two groups.

### 2.7. Randomization and Allocation Concealment

Qualified subjects were randomly assigned to the AET and AET + ISP groups using the random group generator function contained in Microsoft Excel (Microsoft Corp., Redmond, WA, USA) [47]. The subjects, coach, and data collectors were unaware of the group allocation.

### 2.8. Data Analysis

Statistical analyses were performed with SPSS (Ver. 22, SPSS, IL). All results are presented as the mean ± standard deviation (SD). Shapiro–Wilk test and Levene’s test were used to check the assumption of normality and homogeneity of variance, respectively. Differences in the baseline for body composition, anthropometric characteristics, and physical fitness were analyzed using independent sample *t*-test (normally distributed data) and Mann–Whitney U test (non-normally distributed data), between the AET and AET + ISP groups. The percent improvements for the body composition, anthropometric characteristics, and physical fitness were calculated (i.e., 100% × (after − before)/before) [48]. The differences in the percent improvements of variates between two groups were compared applying parametric ANCOVA and non-parametric ANCOVA (Quade’s). With the ANCOVA, the age was regarded as a covariate, and the homogeneity of the within-group regression coefficient was tested before proceeding with parametric ANCOVA. A Post hoc analysis was conducted using the Bonferroni test, and the statistical significance level was set at *p* < 0.05. The effect sizes were calculated using Cohen’s *d* coefficient to reflect the magnitude of between-group difference in percent improvements for various variables, where an effect size of <0.2 indicates trivial difference, 0.2~0.5 is considered a small difference, 0.5~0.8 represents a moderate difference, and >0.8 denotes a large difference [49].

## 3. Results

**Figure 1:**
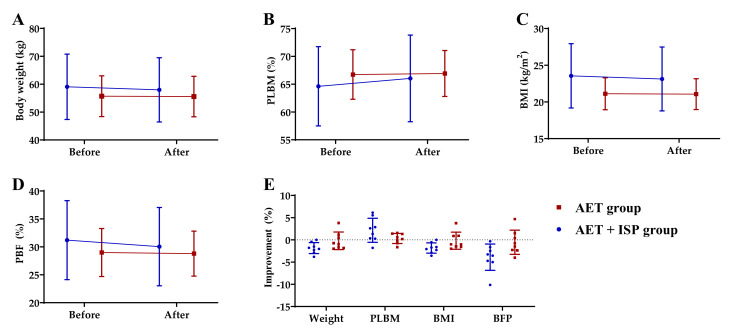
The changes in (**A**) body weight, (**B**) PLBM, (**C**) BMI, and (**D**) PBF for the AET and AET + ISP groups before and after intervention; (**E**) the improvement in body composition of the two groups, shown as a percentage, means the reductions in value for body weight, BMI and PBF, as well as the gain in value for PLBM.

All the subjects (age: 36.13 ± 5.76 years; height: 160.25 ± 4.92 cm) exceeded their target intensity of the AET (RPE = 15 ± 1; HRmax = 163 ± 12) [32], and they reached this intensity consistently in 8 weeks. Table 3 lists the body composition outcomes before and after 8 weeks of intervention for the AET and AET + ISP groups. The changes and percent improvements of the two groups in the body composition are shown in Figure 1. After the 8 week intervention, the body weight, BMI, and PBF of participants in the AET + ISP group decreased by 1.83%, 1.78%, and 3.72%, respectively, and PLBM increased by 2.20%. In the AET group, the body weight, BMI, and PBF decreased by 0.23%, 0.24%, and 0.69%, respectively, and PLBM increased by 0.28%. The effect sizes of between-group differences for the body weight (0.99), PLBM (0.89), BMI (1.04), and PBF (1.18) were large, suggesting the improvements in body composition were greater for the AET + ISP group than the AET group (*p* < 0.05).

**Figure 2:**
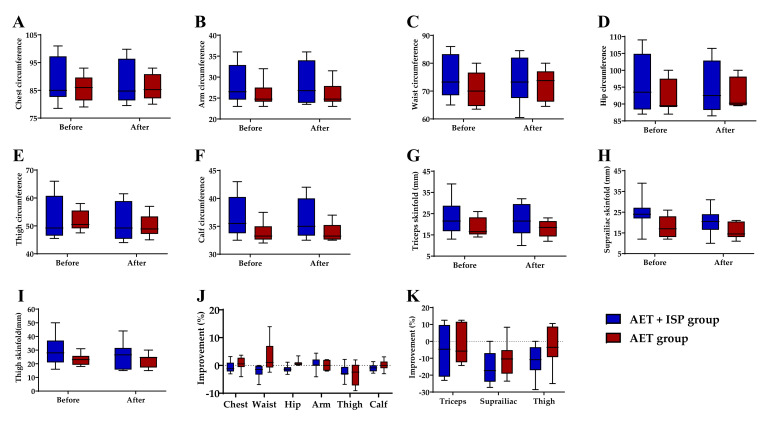
Changes in various body sites, as indicated, for the AET and AET + ISP groups before and after 8 weeks of intervention: (**A**–**F**) circumference changes (cm); (**G**–**I**) thickness of skinfold changes (mm); (**J**) improvements in the circumference (%); (**K**) improvements in the thickness of skinfold (%). The improvement in anthropometric characteristics means the reductions in value for circumference and thickness of skinfold.

The average circumferences and skinfold thicknesses of various body sites before and after 8 weeks of intervention for the AET and AET + ISP groups are shown in Table 4. Figure 2 illustrates the changes and percent improvements in the anthropometric characteristics of the two groups. After the 8-week intervention, the waist and hip circumferences and thigh skinfold thickness of participants in the AET + ISP group decreased by 2.00%, 1.37%, and 11.45%, respectively. In the AET group, the waist and hip circumferences increased by 2.77% and 0.88%, respectively, whereas thigh skinfold thickness decreased by 2.16%. The statistical analysis revealed that the changes in the waist (effect size = 1.19) and hip (effect size = 1.84) were different between groups, with the AET + ISP group showing greater improvements (*p* < 0.05). No significant differences were observed in the improvements of the chest, arm, thigh, and calf circumference, or the skinfold thickness (triceps, suprailiac, and thigh) between the AET and AET + ISP groups (*p* > 0.05).

**Figure 3:**
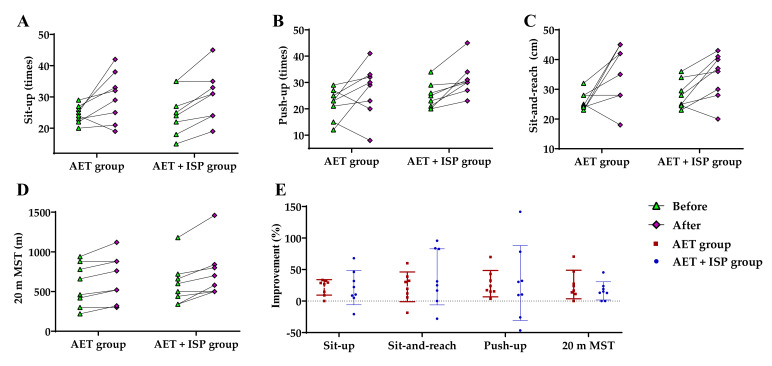
(**A**–**D**) The changes in physical fitness for the AET and AET + ISP groups before and after 8 weeks of intervention using various tests, as indicated; (**E**) the percent improvements of the two groups in the physical fitness means the percent gains in value for each test.

Table 5 summarizes the results for the physical fitness testing of the AET and AET + ISP groups before and after the 8 weeks of intervention. Figure 3 shows the changes and percent improvements in physical fitness for the two groups. After the 8 week intervention, the distance covered in the 20 m shuttle runs by the AET and AET + ISP groups increased by 13.72% and 22.91%, respectively. However, there were no significant between-group differences in the change of 20 m MST, signifying that the AET + ISP group showed similar outcomes to the AET group for improving the cardiopulmonary function and exercise endurance (*p* > 0.05).

## 4. Discussion

ISP is a high-quality, plant-based protein that provides indispensable essential amino acids for protein synthesis in the body [2]. Many previous studies have confirmed that soy protein consumption after strength or resistance exercise training can improve muscle protein synthesis [36], attenuate muscle damage, enhance muscle recovery [35], and promote muscle mass gains [2], but there are few studies concerning the effects of ISP supplementation post AET on health promotion specifically for women. Therefore, this study evaluated the effects of ISP supplementation combined with AET on body composition, anthropometric characteristics, and cardiopulmonary endurance in women. The results of the present study tend to support our hypotheses regarding the positive effects of ISP supplementation since the AET + ISP group were observed to have greater improvements in body composition for all parameters (body weight, PLBM, BMI, and PBF, all effect sizes >0.8), compared with the AET group. Large effect sizes in between-group differences for the circumferences (waist and hip) suggest that the improvements in anthropometric characteristics were also greater for the AET + ISP group, compared with the AET group. For the cardiopulmonary endurance, there was no significant difference between the two groups. Therefore, ISP may be an effective supplementation in women following AET for health promotion and body shape improvement.

Adequate dietary protein supplementation in exercise activities is important for women’s health and improving their body composition. Previous studies in animals and humans showed that a combination of AET and whey protein lead to greater improvements in body composition than AET alone [38,50]. However, all these studies used animal proteins. Due to the positive implications for health and environmental sustainability of plant intake (compared with animal intake), the consumption of plant proteins by exercising individuals has been increasing [4]. ISP is completely plant-based protein source, rich in essential amino acids and with a nutritional value equivalent to that of animal protein [7]. According to previous studies, there was a tendency toward more favorable changes in body composition resulting from ISP supplementation with AET (90–120 min/week for 8 weeks) compared with the AET-only program, and this was defined by a combination of the decrease in body weight and fat mass as well as an increase in lean mass [48,51]. The results of our study are consistent with these reports. We found that AET combined with ISP supplementation improved the body weight, BMI, PBF, and PLBM more effectively than AET alone (all effect sizes > 0.8, Table 3), which may be related to the sustained decrease in appetite, enhanced satiety, and energy expenditure, and promotion of lipid metabolism due to the regulation of the expression of sterol regulatory element-binding proteins after ISP intake [7,13,52]. These findings indicate that ISP is an effective post-exercise supplementation that can induce positive improvements in the body composition of women.

There are a limited number of studies concerning the effects on anthropometric characteristics of ISP supplementation in women following AET. Suh and Lee (2005) found that there were no differences in the thickness of triceps, suprailiac, and thigh skinfold between the high-protein diet with exercise and the exercise-only groups after the intervention period of 8 weeks [51]. A meta-analysis stated that ISP can prevent fat accumulation and ameliorate anthropometric indicators in women [52], and our study supports these concepts. The results of this study showed that no significant differences were noticed in the improvements of skinfold thickness between the AET and AET + ISP groups (Table 4). The effect sizes of between-group differences for the changes of waist and hip circumference were large (Table 4), implying that AET combined with ISP led to more improvements in circumferences compared with AET alone. Furthermore, we observed a slight increase in the waist circumference (before: 70.44 ± 6.17 cm; after: 72.39 ± 5.68 cm) among subjects who received the same AET without ISP supplementation, suggesting increased fat deposits. We speculated that these subjects might have consumed foods that were not appropriate, such as snacks or drinks containing high concentrations of sugar or electrolytes post AET, as there were no diet restrictions during the training period. These observations indicated the importance of ISP and nutritional education in health promotion and ameliorating body image for women.

According to ACSM guidelines, flexibility refers to the ability to move a joint through its complete range of motion and is one of the health-related physical fitness components [32]. Therefore, this study performed the sit-and-reach test to assess low back and hamstring flexibility. Our results showed that, after 8 weeks of intervention, the flexibility all increased in the AET (before: 26 ± 3 cm; after: 35 ± 10 cm) and AET + ISP groups (before: 28 ± 5 cm; after: 34 ± 8 cm), with no statistically significant difference in the percent improvement of flexibility between groups (effect size = 0.44, Table 5). These results were consistent with the report of Suh and Lee [51]. They also observed that the flexibility improved in the ISP diet and AET group (before: 15.6 ± 6.8 cm; after: 18.2 ± 6.6 cm) and AET-only group (before: 14.7 ± 8.8 cm; after: 17.2 ± 7.3 cm). However, no differences were observed in the flexibility between the two groups (*p* > 0.05), after the 8-week AET program. These phenomena suggested that ISP potentially could not improve flexibility. The flexibility is joint specific and mainly depends on the distensibility of the joint capsule, adequate warm-up, and muscle viscosity [32], it can be improved by engaging in flexibility exercises. Flexibility exercise is generally carried out after the warm-up or cool-down phase to improve flexibility by stretching-lengthening and loosening muscles and connective tissues. Performing flexibility exercises ≥2–3 day/week, and a total of 60 s of stretching time for each flexibility exercise are recommended by ACSM.

To examine the effectiveness of AET and/or ISP supplementation in improving cardiopulmonary endurance, all subjects underwent a 20 m MST. The result of our study showed that AET and ISP led to no significant difference in the improvement of cardiopulmonary endurance compared with those who undertook AET without ISP supplementation (Table 5), and this finding is in agreement with previous studies. There are several studies indicating rates of muscle protein synthesis and breakdown are highly sensitive to physical activity and dietary intake, while ISP can provide indispensable amino acids needed to repair and rebuild proteins in the body, whereby a greater intake of ISP can attenuate AET-induced muscle damage, stimulate muscle protein synthesis rates, heighten exercise training adaptations, and enhance muscle recovery [4,12,35,53] but may not raise cardiopulmonary endurance [51]. These findings demonstrated the improvement of cardiopulmonary endurance mainly depends on regular AET rather than ISP, supplementing ISP post AET has beneficial effects on health promotion.

Previous reported a relationship between soy protein supplementation and muscular strength [2,36]. They found that a high soy protein diet combined with resistance exercise resulted in significant gains in muscular strength. This observation was consistent with the studies of Josseet et al. (2010) and Pasiakos, McLellan, and Lieberman (2015), suggesting that as the duration, frequency, and volume of resistance training increase, protein supplementation may promote muscle hypertrophy and increase muscle strength [54,55]. We did not observe differences (effect size < 0.2) between the AET and AET + ISP groups in terms of changes in upper body muscular endurance as evaluated by the 1 min push-up test after 8 weeks of intervention (Table 5). Such different results may be due to differences in the mode of exercise training that was evaluated. In summary, the consumption of ISP supplementation in women following AET resulted in more enhanced favorable changes in body composition and anthropometric characteristics compared with women undergoing only AET, while the improvements of cardiopulmonary endurance were not different.

This study has the limitations that men were not included as subjects. In this study, the average age of all women, who were representative of the sedentary population, was 36.13 ± 5.76 years. Therefore, the results cannot be generalized to men. In addition, the effects of the ISP diet following AET on the body composition, anthropometric characteristics, and cardiopulmonary endurance of women were observed only for 8 weeks. After stopping the intervention, the subsequent changes and sustaining time of these effects are unclear. We hope to further investigate the follow-up effects of ISP intervention on the health of the general population in the future.

## 5. Conclusions

It is feasible to combine ISP and AET among women. The results of this study showed that there was a trend for the consumption of ISP following AET to promote greater reductions in body weight, BMI, PBF, circumferences (waist and hip), and greater gains in PLBM in women compared with those who received the same AET without ISP supplementation, with no differences in the improvements of cardiopulmonary endurance. It is hoped that this study can raise awareness among women regarding ISP, so as to promote the development of women’s health and nutrition.

## Figures and Tables

**Table 1:** Nutritional content of the ISP supplement (for the AET + ISP group).

Contents	AET + ISP
	Per Serving (20 g/pack)
Calories	72 kcal
Protein	11.6 g
Fat	0.4 g
Carbohydrate	5.5 g
Sodium	87 mg

**Table 2:** Subjects’ baseline information in the AET and AET + ISP groups.

	AET (N = 8)	AET + ISP (N = 8)	*p*
Age (years)	38.00 ± 5.88	34.25 ± 5.34	0.203 ^†^
Height (cm)	162.25 ± 4.50	158.25 ± 4.74	0.105 ^†^
Body weight (kg)	55.69 ± 7.30	59.06 ± 11.72	0.501 ^†^
BMI (kg/m^2^)	21.11 ± 2.18	23.55 ± 4.38	0.180 ^†^
PLBM (%)	66.74 ± 4.45	64.62 ± 7.13	0.488 ^†^
PBF (%)	28.99 ± 4.30	31.20 ± 7.07	0.462 ^†^
Circumference (cm)			
Chest	85.63 ± 4.71	88.13 ± 8.20	0.467 ^†^
Arm	25.88 ± 2.89	28.13 ± 4.74	0.271 ^†^
Waist	70.44 ± 6.17	75.06 ± 7.76	0.208 ^†^
Hip	91.94 ± 4.97	95.75 ± 8.39	0.370 ^‡^
Thigh	51.63 ± 3.80	52.63 ± 7.92	0.752 ^†^
Calf	33.81 ± 1.81	36.56 ± 3.76	0.083 ^†^
Skinfold thickness (mm)			
Triceps	18.38 ± 4.57	23.13 ± 8.31	0.225 ^‡^
Suprailiac	18.00 ± 5.10	24.44 ± 7.48	0.064 ^†^
Thigh	23.13 ± 4.26	29.25 ± 11.09	0.268 ^‡^
Physical Fitness			
Sit-up test (times)	25 ± 3	25 ± 7	0.823 ^†^
Push-up test (times)	22 ± 6	25 ± 5	0.302 ^†^
Sit-and-reach distance test (cm)	26 ± 3	28 ± 5	0.295 ^†^
20 m MST (m)	583 ± 271	598 ± 274	0.914 ^†^

Values are presented as the mean ± SD. BMI, body mass index. PLBM, percentage lean body mass. PBF, percentage body fat. ^†^, *p* values are from the independent sample *t*-test. ^‡^, *p* values are from the Mann–Whitney U test.

**Table 3:** The body composition of AET and AET + ISP groups before and after 8 weeks of intervention.

	AET			AET + ISP			*p*	Effect Size
	Before	After	Change	Before	After	Change		
Body weight (kg)	55.69 ± 7.30	55.56 ± 7.25	−0.23%	59.06 ± 11.72	57.98 ± 11.52	−1.83%	0.043 ^†^	0.99
PLBM (%)	66.74 ± 4.45	66.93 ± 4.13	0.28%	64.62 ± 7.13	66.04 ± 7.79	2.20%	0.043 ^‡^	0.89
BMI (kg/m^2^)	21.11 ± 2.18	21.06 ± 2.10	−0.24%	23.55 ± 4.38	23.13 ± 4.35	−1.78%	0.035 ^†^	1.04
PBF (%)	28.99 ± 4.30	28.79 ± 4.02	−0.69%	31.20 ± 7.07	30.04 ± 7.01	−3.72%	0.026 ^†^	1.18

Values are presented as the mean ± SD. BMI, body mass index; PBF, percentage body fat; PLBM, percentage lean body mass. ^†^, *p* values are from parametric ANCOVA. ^‡^, *p* values are from the Quade’s ANCOVA.

**Table 4:** The anthropometric characteristics of AET and AET + ISP groups before and after 8 weeks of intervention.

	AET			AET + ISP			*p*	Effect Size
	Before	After	Change	Before	After	Change		
Circumference (cm)								
Chest	85.63 ± 4.71	86.04 ± 4.71	0.48%	88.13 ± 8.20	87.60 ± 7.80	−0.60%	0.342 ^†^	0.47
Arm	25.88 ± 2.89	25.88 ± 2.80	0.00%	28.13 ± 4.74	28.31 ± 5.04	0.64%	0.877 ^†^	0.24
Waist	70.44 ± 6.17	72.39 ± 5.68	2.77%	75.06 ± 7.76	73.56 ± 8.33	−2.00%	0.025 ^†^	1.19
Hip	91.94 ± 4.97	92.75 ± 4.57	0.88%	95.75 ± 8.39	94.44 ± 7.66	−1.37%	<0.001 ^†^	1.84
Thigh	51.63 ± 3.80	49.98 ± 4.01	−3.20%	52.63 ± 7.92	51.25 ± 6.83	−2.62%	0.665 ^†^	0.23
Calf	33.81 ± 1.81	33.85 ± 1.65	0.12%	36.56 ± 3.76	36.23 ± 3.54	−0.90%	0.268 ^†^	0.64
Skinfold thickness (mm)								
Triceps	18.38 ± 4.57	17.88 ± 3.91	−2.72%	23.13 ± 8.31	21.75 ± 7.55	−5.97%	0.158 ^‡^	0.24
Suprailiac	18.00 ± 5.10	15.88 ± 3.98	−11.78%	24.44 ± 7.48	20.51 ± 6.23	−16.08%	0.297 ^†^	0.53
Thigh	23.13 ± 4.26	22.63 ± 5.04	−2.16%	29.25 ± 11.09	25.90 ± 9.92	−11.45%	0.107 ^†^	0.85

Values are presented as the mean ± SD. ^†^, *p* values are from parametric ANCOVA. ^‡^, *p* values are from the Quade’s ANCOVA.

**Table 5:** Physical fitness of the AET and AET + ISP groups before and after 8 weeks of intervention.

	AET			AET + ISP			*p* ^†^	Effect Size
	Before	After	Change	Before	After	Change		
Sit-up test (times)	25 ± 3	30 ± 8	20.00%	25 ± 7	30 ± 8	20.00%	0.921	0.01
Push-up test (times)	22 ± 6	27 ± 10	22.73%	25 ± 5	31 ± 6	24.00%	0.665	0.03
Sit-and-reach distance test (cm)	26 ± 3	35 ± 10	34.62%	28 ± 5	34 ± 8	21.43%	0.309	0.44
20 m MST (m)	583 ± 271	663 ± 293	13.72%	598 ± 274	735 ± 323	22.91%	0.216	0.53

Values are presented as the mean ± SD. ^†^, *p* values are from parametric ANCOVA.

## Data Availability

The data presented in this study are available on request from the corresponding author.
